# *KEAP1/NRF2* Mutations in Stem Cells Define an Aggressive Subset of Head and Neck Cancer Patients Who Have a Poor Prognosis, Lung Metastasis, and Therapeutic Failure

**DOI:** 10.3390/cancers15205006

**Published:** 2023-10-16

**Authors:** Syed S. Islam, Bedri Karakas, Abdelilah Aboussekhra, Abu Shadat M. Noman

**Affiliations:** 1 Department Molecular Oncology, King Faisal Specialist Hospital and Research Centre, Riyadh 11211, Saudi Arabia; 2 Faculty of Medicine, Al-Faisal University, Riyadh 11533, Saudi Arabia; 3 3 B & B Bio, 4 Professional Drive, Gaithersburg, MD 20879, USA; bedrii@gmail.com; 4 Department Biochemistry and Molecular Biology, The University of Chittagong, Chittagong 4331, Bangladesh

**Keywords:** *Keap1*, *Nrf2*, fast progressors, head and neck cancer, HN-CSCs

## Abstract

**Simple Summary:**

Mutations in head and neck cancer result in abnormal tumor cell growth, increase the risk of distant metastasis, and lead to therapeutic failure. Progenitor cells, head and neck cancer stem cells, and bulk tumor cells may all harbor these mutations, and identifying the mutations in stem cells may facilitate stratifying patients who are likely to benefit from treatment. Patients with head and neck cancer whose tumor exhibits *Keap1*/*Nrf2* mutations in their stem cells are significantly more likely to develop rapid lung metastasis and fail treatment. Our findings suggest a molecular genotyping of head and neck cancer stem cells, which may facilitate personalized treatment strategies and assist in identifying patients who would benefit from chemotherapy, radiotherapy, and targeted therapies.

**Abstract:**

Mutations in *Keap1*/*Nrf2* in head and neck cancer result in abnormal cell growth. Progenitor cells, bulk tumor cells, and head and neck cancer stem cells (HN-CSCs) may all harbor these mutations. Nevertheless, whether *Keap1/Nrf2* mutations in HN-CSCs have an impact on clinical outcomes is unknown. Cancerous HN-CSCs and benign stem cells were obtained from freshly resected head and neck cancer patients (n = 50) via flow cytometry cell sorting and tested for *Keap1*/*Nrf2* mutations. The existence of *Keap1*/*Nrf2* mutations in HN-CSCs, as well as their correlations with tumor mutations, pathologic tumor stage, tumor histologic grades, lung metastasis, treatment outcomes, and the patient’s age and conditions, are assessed at the last follow-up visit. Thirteen tumors were found to have *Keap1/Nrf2* mutations in their HN-CSCs. More than half of the lung metastases and disease progression occurred in HN-CSCs with mutations. Patients whose tumors carried *Keap1/Nrf2* mutations in their HN-CSCs had significantly shorter progression-free survival, overall survival, and time of treatment failure than their non-HN-CSC counterparts. These associations were partly driven by HN-CSCs, in which *Keap1/Nrf2* mutations were overrepresented in fast progressors and associated with an increased risk of disease progression. Our findings suggest that molecular genotyping of HN-CSCs may facilitate personalized treatment strategies and assist in identifying patients who are likely to benefit from chemotherapy.

## 1. Introduction

The head and neck cancer stem cells (HN-CSCs) theory suggests that the malignant cells within a tumor are central for differentiation, proliferation, self-renewal, and distant tumor initiation during metastasis. All of these features of HN-CSC determine the outcome of patients’ survival and progression-free survival as well as their response to therapies [1]. Despite significant advancements in the treatment of head and neck cancer, many patients continue to fail therapy, resulting in disease progression, recurrence, shorter overall survival, and disease-free survival. Over the last decades, many hypotheses have emerged in tandem to shed light on the underlying mechanisms of tumor heterogeneity and the links between these mechanisms and treatment resistance.

To date, several putative cancer stem cell (CSC) markers have been identified in head and neck cancer, including CD44 [2], CD133 [3], CD10 [4], and CD98 [5]. These markers resemble those found in breast and brain cancer. In addition, the aldehyde dehydrogenase (ALDH) activity [6] and side population (SP) [7] have also been identified as potential members of HN-CSCs with enhanced tumorigenic potential and reduced sensitivity to chemo and radiotherapy. The population in head and neck tumors, whether malignant or benign, has been reported to be heterogeneous and to comprise a mixture of genetically distinct positively and negatively expressing CD133, CD44, CD24, and CD49f subclones [8].

So far, there have been no reports of oncogenic dysregulation of stem or progenitor cells in head and neck tumors. Head and neck cancer, on the other hand, is characterized by TP53 mutations and increased genomic disruption due to whole genome duplication, which affects the cell cycle and the PI3K-AKT signaling pathway [9,10]. Apart from these, several oncogenes in the *Keap1/Nrf2* signaling pathway have been identified in head and neck cancer [11]. Additionally, it has been shown that *Nrf2* regulates the maintenance of hematopoietic stem cell functions [12].

The *Keap1-Nrf2* pathway is implicated in the stress response pathway and is responsible for the defense of cells from electrophilic, toxic, and oxidative stress [13]. Furthermore, the *Keap1/Nrf2* pathway is also implicated in chemotherapeutic resistance, growth, proliferation, and elevated CSC population [14,15]. *Keap1* mutations induce *Nrf2*, which in turn acquires malignancy and plays a pivotal role in the development of chemoresistance [16]. *Keap1* and *Nrf2* pathway mutations and their chemoresistance have been reported in lung cancer [17], as well as in head and neck cancer [14,18]. *Keap1* mutations enhance the nuclear accumulation of *Nrf2,* resulting in elevated anti-oxidant stress enzymes and drug efflux pumps [19]. The Cancer Genomic Atlas (TCGA) project has profiled a broad array of somatic genomic alterations in head and neck cancer. Although mutations in the *Keap1/Nrf2* pathway have been reported in 4–5% of head and neck cancer primary tumors [14,18], their existence in HN-CSCs and possible clinical implications have not been investigated.

In this study, we set out to explore the impact of *Keap1/Nrf2* mutations in HN-CSCs and assess the differences in tumor behaviors and clinical outcomes in head and neck cancer. To address this, we explored the hypothesis that *Keap1/Nrf2* mutations in head and neck stem and progenitor cells were linked to frequent lung metastasis, aggressive tumor behavior, and failure of chemoradiation, and in some cases, targeted therapy.

## 2. Materials and Methods

### 2.1. Patients’ Informed Consent, Tissue Collection, and Processing

This study was reviewed and approved by the ethical committee for the Bangladesh Medical Research Council (BMRC # BMRC/NREC/2013-2016/14(1)), and the King Faisal Specialist Hospital and Research Center (KFSH&RC, RAC#2210002). Written informed consent was obtained from all patients according to institutional guidelines. Parts (at least 1.0–1.5 g) of solid head and neck cancer tumors were collected at the time of surgery and for diagnostic requirements without a history of adjuvant therapy. Tissues/samples were transported to the laboratory in a transport medium consisting of an epithelial growth medium, 10% fetal bovine serum, and 1% antibiotics/antimycotics (Invitrogen, Carlsbad, CA, USA). Two blinded pathologists evaluated the tissues for confirmation of head and neck carcinoma. All tissues were minced into small pieces and cryopreserved at −80 °C for future use. Minced tissues were thawed, centrifuged to remove the freezing medium, and enzymatically dissociated using epithelial cell culture medium supplemented with trypsin-EDTA (Invitrogen, Carlsbad, CA, USA) and 2.5 mg/mL of dispase (Roche, Indianapolis, IN, USA).

### 2.2. Flow Cytometry and Cell Sorting

A single-cell suspension was collected by centrifugation, washed in HBSS/2% HICS, and counted to confirm that there were enough cells for FACS sorting. Cell populations were separated according to a previously published method [20]. Briefly, before the cells were labeled with fluorochrome-conjugated antibodies, they were tested for viability with trypan blue and counted to make sure there were sufficient viable cells. First, cells were labeled with fluorochrome-conjugated monoclonal antibodies against CD45 (leucocyte common antigen) and CD31 (a marker of angiogenesis) (fluorescin isothiocyanate conjugated), CD24 (phycoerythrin), CD49f (phycoerythrin-cyanine 5), and CD44 (phycoerythrin cyanine 7). No nonspecific binding was detected when testing for isotype control. Following that, subpopulations were separated based on surface antibody labeling and collected by a sequential discriminatory gating strategy. The CD45*^pos^* and CD31*^pos^* endothelial cells and leukocytes were depleted, leaving only CD24*^pos^*/CD49f*^pos^*, CD24*^neg^*/CD49f*^pos^*, CD24*^pos^*/CD49f*^neg^*, and CD24*^neg^*/CD49f*^neg^* lineage negative cells. CD44 expression frequency was evaluated for these lineage cell populations in all patients. The sorted cells were centrifuged at 680× *g* for 15 min at 4 °C and then prepared for Sanger sequencing. Following tissue analysis, patients’ medical and demographic records were checked and reviewed, and where necessary, additional patient data were collected retrospectively. For each patient, the following key data were retrieved and compiled: age, sex, tumor histology, HPV status, metastatic status, organ with metastases, histologic grade, stages, and time of the last follow-up contact.

### 2.3. Genomic DNA Extraction, PCR Amplification, and Sanger Sequencing

Genomic DNA was extracted from sorted cells (total patient samples: 50) using the PureLink Genomic DNA Kit (Invitrogen, Carlsbad, CA, USA) according to the user manual included with the kit. Genomic DNA was extracted after a 10 min incubation at 55 °C by digestion with Proteinase K and RNase A in the lysis buffer. Following that, ethanol was added, and samples were run through the PureLink Spin column. Centrifugation was performed to allow DNA to bind to the column, which was then washed and cleaned. The purity of the extracted DNA was measured by spectrophotometric determination of the *A_260/280_* ratio, while the concentration was calculated according to the A_260_. PCR amplification and Sanger sequencing were carried out as described previously [14]. Briefly, the coding regions of *Keap1* and *Nrf2* genes were PCR-amplified using 20 ng of DNA-specific primers containing M13 tail sequences in a 25 μL reaction buffer. Supplementary Table S1 contains the sequences for all of the primers. To generate a sufficient amount of DNA for Sanger sequencing reactions, nested PCR amplifications were performed on samples that did not have sufficient DNA. High-fidelity Taq polymerase was used for PCR amplification to avoid errors during the amplification reactions. To rule out any PCR-related artifacts, samples containing mutations were repeated through the PCR amplifications and sequencing steps. For bidirectional sequencing, M13 forward and reverse primers were used.

### 2.4. Cell Lines, Cell Cultures, and Cell Viability Assay

We used SSC9, Cal33, and freshly resected patients’ tumor cells. The cells were grown in DMEM/F12 medium supplemented with 10% and 20% fetal bovine serum. Cells were seeded in 96-well plates at a density of 2000 cells per well for dose–response and IC50 experiments. The cells were incubated with cisplatin and CB-839 for 96 h. Cell viability was determined by the AlamarBlue assay using the manufacturer’s instructions. The fluorescence was measured using a SPECTRAmax Gemini Spectrophotometer (540 nm excitation and 590 nm emission) after 4 h of incubation with AlamarBlue reagent (10% of the total volume). The GRmetrics package was used to determine the IC50, and the dose–response curves were generated using R statistical software (version 4.1.0).

### 2.5. Sphere Formation Assay

FACS-sorted cells were cultured in 6-well ultra-low attachment plates at a density of 1000 cells per well in a growth factor-supplemented CSC medium. The growth and size of the spheres were monitored and recorded for 5–7 days. Sphere-forming efficiency was calculated as the number of actual spheres/number of cells plated × 100. Spheres > 50 μm were counted manually.

### 2.6. TCGA and HNC-MSKCC Public Data Sets

Data related to TCGA (The Cancer Genomic Atlas Research) and MSKCC studies were obtained from cBioPortal (available at: http://cbioportal.org; accessed on 27 August 2022). Mutation and clinical data for primary (N = 512) and metastatic (N = 134) biopsies were retrieved from the HNSCC Firehose legacy cohort. The group of HNSCC with a mutant was defined by the presence of somatic mutations in either *Keap1* or *NRF2*. Fast progressors (FPs), conventional progressors (CPs), and slow progressors (SPs) were determined based on progression-free survival (PFS) tertiles [21]. Considering the size of our study cohort, we further analyzed the enrichment of *Keap1/Nrf2* mutations across PFS quartiles.

### 2.7. Statistical Analysis

The Pearson Chi-square test of independence (two-tailed) and/or the Fisher exact test were used to compare differences in clinical characteristics between categorical variables. To compare parametric data between the two groups, the Student’s *t*-test was used. Survival curves were estimated with Kaplan–Meier and compared using the log-rank test. Multivariate Cox models for PFS and overall survival (OS) were built with variables containing confounding factors. The related estimates were reported as the hazard ratio (HR) and 95% confidence interval (CI). The level of significance was determined by a *p*-value less than 0.05. For statistical analysis, R statistical software (version 4.1.0) was used.

## 3. Results

### 3.1. Keap1/Nrf2 Mutation Analysis in HN-CSCs

We identified 50 patients with head and neck cancer who were treated for curative intent with conventional chemo, radiotherapy, or targeted therapy. Patients’ tumor characteristics, age, HPV status, histology, and tumor mutations are summarized in Table 1; Supplementary Table S2. The mean interval between disease diagnosis and follow-up time was 28 months. The most common site of metastasis was the lung, and 11 patients (22%) had lung metastases. The median time between initial disease diagnosis and development of lung metastasis was 12.7 months (range: 5.2–17.6). There was no correlation between tumor size and lung metastasis (*p* = 0.105). The majority of patients with lung metastasis displayed no symptoms, and lung metastasis was mainly detected by chest computer tomography (CT).

We found a higher representation and frequency of *Keap1/Nrf2* mutations in HN-CSCs than in their non-CSC counterparts. Among the 50 tumors, 13 patients (26%) had HN-CSCs *Keap1/Nrf2* mutations, 9 of which are *Keap1*, and the remaining tumors were *Nrf2* (Table 2; Supplementary Table S2). 11 were particularly detected in the HN-CSC group, while only 2 *Keap1* mutations were detected in the non-CSC group. Tumor cells with high CD44 expression were first reported in HN-CSC in head and neck squamous cell carcinoma [2] with a varying degree of expression intensity. Due to the frequent expression variation of CD44 in the HN-CSC population, a subset of the tumors was particularly assessed for CD44-positivity. In the CD24*^pos^*/CD49f*^pos^* sorted cell population, 40 out of 48 patients’ tumor cells had more than 80% of the cells positive for CD44. Interestingly, the CD44 expression pattern was variable among patients in the CD24*^neg^*/CD49f*^pos^*, CD24*^pos^*/CD49f*^neg^*, and CD24*^neg^*/CD49f*^neg^* cell populations. The mean variability in these groups was detected at 55%, (range, 15% [6 of 40 patients]) to 82% (33%, [13 of 40 patients]) of cells being positive for CD44. We then assessed how CD44 expression differed between head and neck cancer stem and progenitor cells with and without mutations. No significant expression differences in CD44 were detected between the two groups.

**Figure 1:**
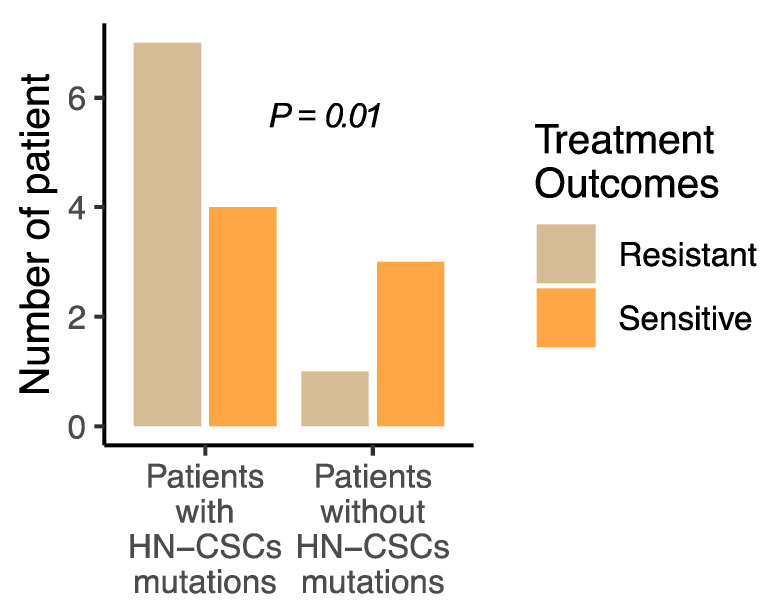
*Keap1/Nrf2* mutations in HN-CSC confer head and neck cancer treatment resistance. The number of treatment-resistant patients is higher in tumors carrying *Keap1/Nrf2* mutations in HN-CSC compared with tumors without HN-CSC mutations (*p* = 0.01).

Next, we assessed the association between the presence of these mutations (*Keap1/Nrf2*) in HN-CSCs and patients’ clinical variables. No statistically significant associations were achieved with patients’ age and tumor size (Table 2). However, a statistically significant correlation was observed between the existence of mutations in HN-CSCs and disease progression (*p* = 0.04), lung metastasis (*p* < 0.01), and lymph node positivity (*p* < 0.001) (Table 2). All patients with *Keap1/Nrf2* mutations in HN-CSCs received a line of chemotherapy, while only four patients with tumors without mutations in HN-CSCs received chemotherapy. Seven of eleven patients with HN-CSC mutations continued to experience disease progression even after undergoing chemoradiotherapy and targeted therapy. Following the detection of lung metastasis in patients with a mutation in the HN-CSC group, three patients died during the follow-up period. Moreover, statistically significant differences were observed between the existence of HN-CSC mutations and treatment outcomes (Figure 1; *p* < 0.01). Importantly, this significance was merely higher when lymph node (*p* < 0.001) positivity and lung metastasis (*p* < 0.001) were included (Table 1).

### 3.2. Keap1/Nrf2 Mutations in HN-CSCs Are Associated with Inferior Survival Outcomes

**Figure 2:**
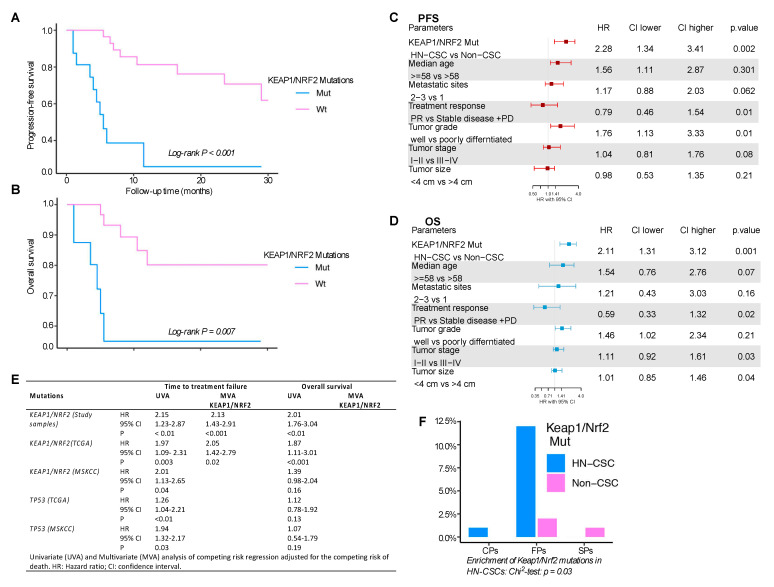
*Keap1/Nrf2* mutations in HN-CSCs are associated with inferior survival outcomes. (**A**,**B**) Kaplan–Meier survival curves of progression-free survival (PFS) (**A**) and overall survival (OS) (**B**), comparing *Keap1/Nrf2* in HN-CSCs and non-CSCs counterparts. (**C**,**D**) Forest plot illustrating the multivariate (MVA) Cox regression analysis of PFS and OS. (**E**) Univariate (UVA) and multivariate (MVA) comparing the risk regression analysis adjusted for the confounding risk of death. (**F**) Bar graph summarizing the distribution of mutations investigated across two different populations (HN-CSCs and non-CSCs). CFs—Conventional progressors, FPs—Fast progressors, SPs—Slow progressors. (Chi-square test *p* = 0.03). HR, hazard ratio; CI, confidence interval; TCGA, The Cancer Genome Atlas; MSKCC, Memorial Sloan Kettering Cancer Center; PD, progressive disease; PR, partial response; HN-CSCs, head and neck cancer stem cells.

We next investigated whether *Keap1/Nrf2* mutations in HN-CSCs had any adverse impact on progression-free survival (PFS) and overall survival (OS). Patients whose tumors harbored *Keap1/Nrf2* mutations in HN-CSCs had significantly shorter PFS (log-rank *p* < 0.001) and OS (log-rank *p* = 0.007) than the patients with non-CSC disease (Figure 2A,B). Univariate Cox models showed that patients with *Keap1/Nrf2*-mutant genes were at an increased risk of disease progression and death. Moreover, multivariate Cox regression models indicated that *Keap1/Nrf2* mutations in HN-CSCs are independent predictors of adverse survival outcomes compared to the non-CSC group (PFS, HR = 2.28, 95% CI: 1.34–3.41, *p* = 0.002; OS, HR = 2.11, 95% CI: 1.31–3.12, *p* < 0.001) (Figure 2C,D).

We then analyzed the time of treatment failure (TTF) and OS associations with the TP53 gene, which are frequently mutated in head and neck cancer tumors, from the TCGA and MSKCC data sets. A univariate Cox regression analysis was performed on *Keap1/Nrf2* and TP53 mutations. TP53 mutations from the TCGA and MSKCC cohorts were negatively associated with TTF on univariate analysis (TCGA cohort, HR = 1.26, 95% CI: 1.04–2.21, *p* < 0.01; MSKCC cohort, HR = 1.94, 95% CI: 1.32–2.17, *p* = 0.03) (Figure 2E). On the other hand, *Keap1/Nrf2* mutations in HN-CSC were significant predictors of TTF and OS in our study samples (TTF, HR = 2.13, 95% CI: 1.43–2.91, *p* < 0.001), as well as TCGA (TTF HR = 2.05, 95% CI: 1.42–2.79, *p* = 0.003) and MSKCC cohorts (TTF HR = 2.01, 95% CI: 1.13–2.65, *p* = 0.04) (Figure 2E). Thus, *Keap1/Nrf2* pathway mutations in HN-CSC are strongly associated with poor outcomes with treatment failure in head and neck cancer.

To better understand the clinical significance of *Keap1/Nrf2* mutations in HN-CSCs, we verified whether *Keap1/Nrf2* alterations displayed a differential disease outcome in three disease progression settings, namely fast progressors (FPs), conventional progressors (CPs), and slow progressors (SPs). These groups were defined based on PFS (see materials and methods). Importantly, in HN-CSCs, *Keap1/Nrf2* mutations were significantly overrepresented in the group of FPs (Chi^2^ *p* = 0.03) (Figure 2F), indicating that disease progression is intimately tied with *Keap1/Nrf2* mutations in HN-CSCs.

### 3.3. Keap1/Nrf2 Mutant Cells Experience Therapeutic Resistance and Chemosensitivity to Glutamine Inhibitor

**Figure 3:**
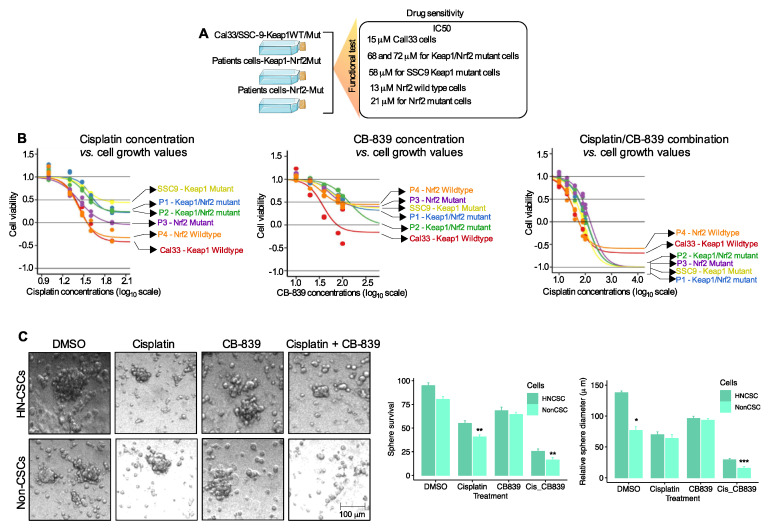
Cell viability of head and neck cancer cells treated with cisplatin and CB-839. (**A**) Schematic diagram for assessing the drug treatment using patient cells and head and neck cancer cell lines (image was produced and modified from Server Medical Art). (**B**) Cell viability was determined in the indicated head and cancer cells with cisplatin, CB-839, and a combination of cisplatin and CB-839. All experiments were performed with triplicated and three independent experiments. (**C**) Relative number of tumorspheres treated with vehicle, cisplatin (10 μM, CB-839 (100 nM), and a combination of both from HN-CSC and non-CSC groups (N = 3 biological replicates). Students *t*-test, * *p* < 0.05; ** *p* < 0.01; *** *p* < 0.001.

Prior studies have demonstrated that a loss of *Keap1* promotes dependence on glutamine metabolism and a sensitivity to glutamine inhibitors [14,22,23]. To investigate whether the presence of *Keap1/Nrf2* mutations might confer sensitivity to the chemotherapy agent, cisplatin, or the small-molecule glutaminase inhibitor, CB-839, to target the glutamine metabolism, we tested a panel of HNSCC cell lines and cells freshly isolated from patients’ tumors during surgery. On a side note, SSC9 is a *Keap1* mutant line, whereas Cal33 is a *Keap1* wild-type. On the other hand, *Keap1/Nrf2* mutant cells were derived from two *Keap1/Nrf2* mutant patients’ tumors who had lung metastases and were also resistant to chemoradiation and targeted therapies. These results yielded IC50 15 μM for Cal33 cells, 68, and 72 μM for two *Keap1/Nrf2* mutant patient-derived cells, 58 μM for SSC9 *Keap1,* and 13 and 21 μM for *Nrf2* wild-type and mutant cells (Figure 3A,B). From these results, there appears to be a distinct difference in the treatment outcome with cisplatin alone, nevertheless, cells with either *Keap1* mutations or *Keap1/Nrf2* mutations exhibit increased resistance when compared to *Keap1* or *Nrf2* wild-type cells (Figure 3B). To identify a potential approach for chemosensitizing *Keap1* mutant or *Keap1/Nrf2* mutant cells, we used CB-839, a small-molecule glutaminase inhibitor. CB-839 exhibits significant antiproliferative activity in several types of cancer and xenografts, such as triple-negative breast cancer [24], lung cancer [22,25], head and neck cancer [14], and lymphoma cancer [26]. We tested the single treatment of CB-839 and a combination of cisplatin in *Keap1* wild-type, *Keap1* mutant, and *Keap1/Nrf2* dual mutant cells. CB-839 treatment alone had minimal sensitivity in the *Keap1* or *Keap1/Nrf2* mutant cells; *Keap1* wild-type Cal33 cells showed modest sensitivity in a range of 5–100 nM doses (Figure 3B). Interestingly, a combination of cisplatin and CB-839 preferentially killed *Keap1* wild-type (Cal33), *Keap1* mutant (SSC9), and *Keap1/Nrf2* mutant patients’ tumor cells (Figure 3B). These results imply that CB-839 chemosensitizes *Keap1* mutant or *Keap1/Nrf2* mutant cells.

Finally, we tested the resistance and sensitivity to the combination of cisplatin and CB-839 in patients’ cells that were identified as having *Keap1/Nrf2* mutations in their HN-CSCs. In vitro, tumorsphere assays demonstrate that *Keap1/Nrf2* mutant cells are significantly more resistant than non-CSC cells. However, combination treatment with cisplatin and CB-839 greatly reduced tumorsphere formation (Figure 3C). These results suggest that the combination of cisplatin and CB-839 may have superior sensitivity to *Keap1/Nrf2* mutant HN-CSCs.

## 4. Discussion

Head and neck cancer lung metastasis cannot be easily defined because the complex lymphatic network occurs with variable frequencies depending on the metastatic sites, T-stage, tumor size, and histological characteristics of the primary lesion. In this study, we present evidence of the clinical ramifications, with a particular emphasis on *Keap1/Nrf2* mutations in HN-CSCs. The rationale behind our hypothesis was that patients with head and neck cancer whose tumors carry *Keap1*/*Nrf2* mutations in HN-CSCs are more likely to develop lung metastases. Moreover, patients with metastatic head and neck cancer in which HN-CSCs have mutations have a significantly shorter time to treatment failure and overall survival. It is unclear if the frequent deregulation of *Keap1/Nrf2* mutations in HN-CSCs can be translated into clinically useful information. Nevertheless, our study reports that *Keap1/Nrf2* mutations in HN-CSCs are an independent predictor of lung metastasis and a possible shorter time to treatment failure. 

In our case, only 2 of 13 patients (16%) in the tumor without HN-CSC group had lung metastasis but had a better therapeutic response, suggesting that *Keap1/Nrf2* mutations in HN-CSCs may be partially responsible for head and neck cancer lung metastasis and the least therapeutic outcome. However, other mutations, such as Notch1, CDKN2A, FAT1, PTEN HRAS, and PI3KCA, frequently occurring in head and neck cancer, may influence metastatic events [18,27]. In our study, however, there was a significant relationship between tumors with and without HN-CSC groups. 9 of 11 (82%) patients in the HN-CSCs group had lung metastasis, while only 2 of 39 (5%) had lung metastasis without mutation in the HN-CSC group, and only 2 had additional metastatic sites (1 in the bone and 1 the non-cervical node). No additional metastatic spread was found in tumors without the HN-CSC group. Recent studies, including ours, have shown that the *Keap1/Nrf2* pathway contributes to the development and progression of head and neck cancer [14,17]. Given the intrinsic link between the *Keap1/Nrf2* pathway and metastatic possibility, it is likely that metastatic tumors harboring *Keap1/Nrf2* mutations in HN-CSCs may pose multiple risks of distant metastatic disease. Several previous studies have suggested a role for *Keap1* and *Nrf2* in chemo/radio-resistance [28,29], but none have examined and explored the associations between mutations and clinical consequences, particularly in mutations in the HN-CSC population and chemo–radio or even targeted therapy resistance. Our straightforward experimental approaches allowed us to separate the HN-CSC population from tumor tissues and analyze the mutations from the sorted HN-CSC population, which has greater clinical implications given the fact that cancer stem cells within the tumors have a dire impact on therapeutic response and disease recurrence. Our results suggest the critical role of *Keap1/Nrf2* mutations in tumors with enriched HN-CSCs during lung metastasis because a mutation in these genes leads to increased self-renewal of clonal changes of HN-CSCs in the lung, which has not been reported previously and is of clinical relevance. These results suggest that the detection of these mutations in the HN-CSCs is present in the early development of lung metastatic tumors as well as primary head and neck cancer development. In addition, the impact of *Keap1*/*Nrf2* loss in HN-CSCs and tumors is greater and leads to therapeutic failure compared with a loss of either gene alone. Given the high rate of *Keap1/Nrf2* in HN-CSCs, it is likely that once HN-CSCs acquire mutations in *Keap1/Nrf2,* they may defeat the wild-type or resident stem cells. These features develop a group of mutated cells which acquire more rapid growth characteristics and are at a higher risk of acquiring an additional mutation, leading to metastatic tumor formation in distant metastatic sites, particularly in the lung. While the *Keap1/Nrf2* mutations in the HN-CSCs are considered damaging, clinical observation from our findings suggests the most devastating impact on the outcome of patients’ therapeutic failure after chemo-radio-targeted therapy. 

The prognostic significance of *Keap1/Nrf2* signaling pathway mutations in head and neck cancer remains mostly unexplored. Prior studies mostly focused on immunohistochemical analysis of *Keap1* and or *Nrf2* have shown associations of expression with outcomes. One study reports the overexpression of *Keap1*/*Nrf2* without reaching a significantly worse survival rate, while in lung cancer it was found that *Keap1*/*Nrf2* is an independent prognostic factor [17]. From these conflicting reports, we speculate that the variability of the mutations, tumor architecture and heterogeneity, and pathway complexity may contribute to the different outcomes. Recently, we have reported that *Nrf2* overexpression due to *Keap1* alterations had a poor prognosis, overall shorter survival, and therapeutic failure in head and neck cancer [14]. Given the rapid progress in sequence-based tumor genotyping and the data presented here, there is an important element of novelty that holds the potential to open up novel therapeutic strategies, adding to the further molecular segmentation of head and neck cancer. The severity of the disease, metastasis, and therapeutic failure associated with the *Keap1/Nrf2* mutations in the HN-CSCs, as well as being significantly enriched in FPs. Thus, *Keap1/Nrf2* mutations plausibly define a molecular subset of HN-CSC, which is characterized by self-renewal, intrinsic chemotherapy resistance, and incredibly aggressive behavior.

The degree to which the definitive collection and presence of HN-CSCs from fresh tumor tissues, the degree of accurate molecular analysis, and the association of these traits with patients’ clinical outcomes could potentially be used for diagnosis purposes and personalized treatment strategies for patients with metastatic head and neck cancer. The results from this study provide credence to the idea that analysis of both HN-CSCs and the evaluation of the head and neck cancer tumors might be useful for better treatment strategies in patients who harbor *Keap1/Nrf2* mutations in HN-CSCs. Additionally, the presence of *Keap1/Nrf2* mutations in HN-CSCs could potentially be used to select the best therapy or identify patients who may benefit from a particular therapy. 

## 5. Conclusions

As HN-CSCs are important drivers of tumor initiation, progression, and metastasis, analyzing HN-CSCs can likely reveal specific information about tumor growth and metastatic potential that may not be possible to acquire from non-HN-CSC cells. This directs our focus to either target HN-CSCs or co-target HN-CSCs and non-HN-CSCs (bulk tumors) to overcome chemoresistance and achieve maximum clinical success in metastatic head and neck cancer patients. Treatment directed at eradicating HN-CSCs is one of the attractive future directions for improving the clinical outcome of patients as well as new ways for head and neck cancer diagnosis and treatment planning. Obtaining further knowledge on HN-CSC analysis and expanding further research on the characteristics of HN-CSCs can generate precise information on tumor growth and metastatic potential. Finally, we are aware of any potential risks associated with the retrospective design single-center study and smaller sample size. Nevertheless, our conclusions were greatly strengthened by the reproducibility of our findings across two distinct but clinically comparable cohorts.

## Figures and Tables

**Table 1:** Baseline characteristics of head and neck cancer patient (n = 50).

Characteristics		N (%)
Age at diagnosis	Median (IQ range)	59.6 (48.3–72.1)
Sex	Male	41 (82)
	Female	9 (18)
HPV status	Positive	7 (14)
	Negative	43 (86)
Histology	Oral cavity	15 (30)
	Base of tongue	9 (18)
	Oropharyngeal	18 (36)
	Laryngeal	8 (16)
Primary treatment		
	Surgery > XRT with or without chemo	5 (10)
	Concurrent chemo-XRT	11 (22)
	Induction > definitive therapy	13 (26)
	Surgery > XRT > Salvage chemo/XRT	0
	Surgery alone	12 (24)
	XRT alone	9 (18)
Exposure to platinum in metastatic setting		
	No	12 (24)
	Yes	38 (76)
Exposure to cetuximab in a metastatic setting		
	No	31 (62)
	Yes	19 (38)
Performance status		
	0–1	36 (72)
	2	14 (28)

**Table 2:** Patients and tumor characteristics.

Parameters	Tumors with HN-CSC Mutations (n = 13)	Tumors without HN-CSC Mutations (n = 37)	*p*-Value ^a^
Age at diagnosis, y, Median	62.5 (58.1–73.9)	57.1 (54.7–81.3)	0.27 ^b^
Tumor size (cm), Median	3.8 (11.9–4.2)	3.2 (1.6–4.6)	0.10 ^b^
Disease progression, %, (No./total No.)	53 (7/13)	5 (2/37)	0.04
Lung metastasis, % (No./total No.)	69 (9/13)	5 (2/37)	0.001
Lymph nodes	46 (6/13)	5 (2/37)	0.001

HN-CSCs: Head and neck cancer stem cells; ^a^ Fisher exact test, ^b^ unpaired two-tailed *t*-test.

## Data Availability

All data acquired or analyzed during this study are included in this published manuscript (and its Supplementary Information). However, the data can be shared upon reasonable request.

## Supplementary Materials

The following supporting information can be downloaded at: https://www.mdpi.com/article/10.3390/cancers15205006/s1, Table S1: Primers used in PCR amplifications from FFPE tumor sections for Sanger sequencing; Table S2: List of *Keap1* and *Nrf2* mutations detected in 50 head and neck cancer patients.
