# Supplementary material for: KEAP1/NRF2 Mutations in Stem Cells Define an Aggressive Subset of Head and Neck Cancer Patients Who Have a Poor Prognosis, Lung Metastasis, and Therapeutic Failure
Source: Cancers (Basel). 2023 Oct 16;15(20):5006. doi: 10.3390/cancers15205006 (PMC10605399; doi:10.3390/cancers15205006)
Supplement: Supplementary file 1 [file cancers-15-05006-s001.zip › Supplementary Table S1.pdf]

Supplementary Table S1. Primers used in PCR amplifications from FFPE tumor sections for Sanger sequencing.

Primer	Gene	Coding Exon	Sequence	Amplicon size (bp)
KEAP1Ex1F	KEAP1	1	cctcatccagccctgtcttc	303
KEAP1Ex1R			ctcgatcacgtagaagacctt	
KEAP1Ex2-2F	KEAP1	2	caaggactacctggtcaagatc	302
KEAP1Ex2-2R			ggttgtaacagtccagggc	
KEAP1Ex3F	KEAP1	3	tccacgaaggtcagctataatgg	254
KEAP1Ex3R			gcttcggatggtgttcattgc	
KEAP1Ex4F	KEAP1	4	gagtcaccttctctgcatgg	248
KEAP1Ex4R			gatgggctagtcaggactctt	
KEAP1Ex5F	KEAP1	5	gctgcatctctctctttctgtc	239
KEAP1Ex5R			gtacagttctgctggtcaatc	
NRF2Ex2F	NRF2	2	accatcaacagtggcataatgtg	397
NRF2Ex2R			ctgccataactttcccaagaac	
