# Supplementary material for: KEAP1/NRF2 Mutations in Stem Cells Define an Aggressive Subset of Head and Neck Cancer Patients Who Have a Poor Prognosis, Lung Metastasis, and Therapeutic Failure
Source: Cancers (Basel). 2023 Oct 16;15(20):5006. doi: 10.3390/cancers15205006 (PMC10605399; doi:10.3390/cancers15205006)
Supplement: Supplementary file 1 [file cancers-15-05006-s001.zip › Supplementary Table S2.pdf]

Supplementary Table S2: List of *Keap1* and *Nrf2* mutations detected in 50 head and neck cancer patient.						
**Patient no.**	**Tumor size, cm.**	**N-stage**	**T-stage**	***Clinical stage***	***Tumor mutations***	***HPV status***
Patient # 1	4.2	N3	T4	III	c.403C>T *(Keap1)* *With HN-CSC mutation*	-
Patient # 2	3.6	N3	T3	III	c.1111G>A *(Keap1)* *With HN-CSC mutation*	-
Patient # 3	2.8	N2	T3	IV	c.1129G>A *(Keap1)* *With HN-CSC mutation*	-
Patient # 4	3.1	N1	T2	III	c.1766A>G *(Keap1)* *With-HN-CSC mutation*	-
Patient # 5	3.7	N2	T2	III	c.474T>C *(Keap1)* *With HN-CSC mutation*	-
Patient # 6	2.7	N3	T2	IV	c.1152C>T *(Keap1)* *Without HN-CSC mutation*	-
Patient # 7	4.1	N2	T4	IV	c.1413C>G *(Keap1)* *With-HN-CSC mutation*	-
Patient # 8	3.2	N3	T2	IV	c.1161C>T *(Keap1)* *With HN-CSC mutation*	-
Patient # 9	2.2	N2	T3	III	c.1815C>T *(Keap1)* *With HN-CSC mutation*	-
Patient # 10	2.1	N1	T4	IV	c.241G>C *(Nrf2)* *With HN-CSC mutation*	-
Patient # 11	3.4	N1	T2	IV	c.145G>A *(Nrf2))* *Without HN-CSC mutation*	-
Patient # 12	4.6	N3	T3	IV	c.165C>T *(Nrf2))* *With HN-CSC mutation*	-
Patient # 13	2.2	N2	T3	III	c.162G>A *(Nrf2)* *With HN-CSC mutation*	-
Patient # 14	1.5	N0	T1	III	Mutation not detected	-
Patient # 15	2.1	N1	T1	II	Mutation not detected	-
Patient # 16	1.6	N1	T2	III	Mutation not detected	-
Patient # 17	3.1	N2	T1	III	Mutation not detected	-
Patient # 18	1.3	N1	T4	IV	Mutation not detected	-
Patient # 19	1.6	N2	T3	III	Mutation not detected	-
Patient # 20	1.9	N0	T1	IV	Mutation not detected	+
Patient # 21	2.1	N3	T2	IV	Mutation not detected	+
Patient # 22	1.7	N2	T1	IV	Mutation not detected	-
Patient # 23	3.2	N1	T3	III	Mutation not detected	-
Patient # 24	1.5	N0	T1	IV	Mutation not detected	-
Patient # 25	2.5	N2	T1	III	Mutation not detected	-
Patient # 26	1.2	N3	T2	IV	Mutation not detected	+
Patient # 27	2.2	N0	T3	IV	Mutation not detected	-
Patient # 28	3.0	N0	T1	II	Mutation not detected	-
Patient # 29	2.1	N2	T1	III	Mutation not detected	-
Patient # 30	1.2	N0	T2	III	Mutation not detected	-
Patient # 31	1.8	N3	T2	IV	Mutation not detected	-
Patient # 32	2.0	N2	T1	IV	Mutation not detected	-

Patient # 33	2.1	N2	T3	IV	Mutation not detected	-
Patient # 34	3.1	N0	T1	IV	Mutation not detected	+
Patient # 35	2.4	N1	T2	III	Mutation not detected	-
Patient # 36	3.0	N1	T1	IV	Mutation not detected	-
Patient # 37	1.7	N3	T1	IV	Mutation not detected	+
Patient # 38	2.8	N1	T2	III	Mutation not detected	-
Patient # 39	1.1	N1	T2	II	Mutation not detected	-
Patient # 40	2.2	N2	T1	IV	Mutation not detected	+
Patient # 41	2.8	N0	T2	III	Mutation not detected	-
Patient # 42	2.4	N3	T2	III	Mutation not detected	-
Patient # 43	1.2	N2	T2	IV	Mutation not detected	-
Patient # 44	1.0	N2	T3	IV	Mutation not detected	+
Patient # 45	1.7	N0	T1	III	Mutation not detected	+
Patient # 46	2.0	N1	T1	IV	Mutation not detected	+
Patient # 47	1.9	N2	T3	IV	Mutation not detected	+
Patient # 48	4.3	N1	T2	III	Mutation not detected	-
Patient # 49	2.7	N3	T2	IV	Mutation not detected	+
Patient # 50	1.6	N2	T1	III	Mutation not detected	+
